# More pesticides—less children?

**DOI:** 10.1007/s00508-019-01566-z

**Published:** 2019-11-07

**Authors:** Hanns Moshammer, Michael Poteser, Hans-Peter Hutter

**Affiliations:** Department of Environmental Health, Medical University of Vienna, 1090 Vienna, Austria

**Keywords:** Pesticides, Male fertility, Coffee plantation, Farm workers, Non-linear associations

## Abstract

A previously presented study investigated the impact of recent pesticide exposure on cytological signs of genotoxicity and on symptoms of intoxication in 71 male coffee workers in the Dominican Republic. An unexpected finding of this study was that conventional farming workers, among other symptoms, reported fewer children than controls working in organic farms without pesticide use. This study set out to investigate possible reasons for the latter difference. One statistical problem of this analysis is that the age of the workers is a strong predictor for the number of children and available data on the exposure determinants “duration of pesticide exposure” as well as “age at first pesticide exposure” are correlated with age. To correctly control statistics for these confounding parameters, different approaches to best control for age were explored. After careful elimination of the age-related confounding factors, a reduced number of children was still observed in exposed workers. The clearest effect is seen in those workers that reported first exposure before the age of 20 years. Socioeconomic factors could still confound that finding, but a direct effect of early life pesticide exposure is the most likely explanation of the observation.

## Introduction

Male fertility globally is observed to decline [1–3]. This is also reflected in the new World Health Organization (WHO) reference values for human semen characteristics that are lower than previous ones [4]. Lifestyle and environmental factors have been proposed as causal factors, among these especially chemicals disrupting endocrine function. Some pesticides and especially several of the old organochlorine pesticides are suspected to have endocrine disruptive properties and so a connection has been proposed between pesticide use and male fertility problems [5–7].

In a previous report first results were presented [8–10] of the cross-sectional study in coffee plantation workers in the Dominican Republic (D.R.). In the study 38 workers in conventional agriculture with heavy exposure to pesticides were compared to 33 workers in organic farming with no pesticide use for at least 5 years. Although a low-level pesticide exposure, for example by wind drift or by contact with contaminated goods is also likely to affect organic farm workers, the frequency and quantity of intake are expected to be significantly higher in conventional farming. The conventional farming workers are therefore referred to as “professionally exposed group” and the farmworkers of organic farms as “professionally non-exposed”.

In addition to buccal cell sampling to determine indicators of genotoxicity, in a short questionnaire workers were also asked about symptoms commonly associated with pesticide poisoning, about demographics and indicators of socioeconomic status, and possible confounding factors. This survey indicated a clear difference in age and number of children between organic and conventional farmworkers (see Results section). As both parameters are potentially linked to pesticide exposure, which could indicate serious health-related implications, this was investigated in more detail.

It was concluded that the difference in age could be an interesting starting point for further considerations and a more detailed analysis. Several hypotheses are conceivable: workers in organic farming are expected to be, on average, older than their counterparts in conventional farming with professional pesticide exposure: (a) organic farming must rely on targeted biological pest control measures that call for more experienced (and therefore generally older) workers. (b) Organic farming is also often linked to fair-trade schemes and thus might offer better social and occupational conditions. Because the first wave of workers sought better paid jobs at organic farming, the following generation of workers was limited to applying for jobs in conventional farming with pesticide usage. (c) Exposure to pesticides leads to a range of health-related symptoms [9]. To avoid these detrimental effects, workers might tend to switch to organic farming jobs whenever they have the opportunity, causing a thinning out of the older ones in the exposed sector (“healthy worker” effect) [11, 12]. (d) Pesticide workers have a shorter life expectancy due to chronic intoxication.

The number of children was one of several parameters in questions on social status and housing conditions. In a previous paper the differences between the groups were described, but not further investigated. This article provides a more detailed analysis to clarify the difference in the number of children after adjustment for age. Indeed, some pesticides have been implicated in endocrine disturbances [15], disturbances of reproduction [16–18] and adverse effects on the male reproductive system [19–26], but comparative epidemiologic studies in pesticide exposed tropical farmworkers are sparse. Thus, the apparent differences between organic and conventional farmworkers were investigated in detail.

## Material and methods

### Recruitment

Recruitment of workers turned out to be difficult under field conditions in the poorly developed study area. To maximize the number of potential participants, a snowball recruitment scheme was applied. Although this recruitment strategy was found to be feasible, it came with the disadvantage of impossibility for matching ages among participants. Organic and conventional group showed a difference in mean age. As the exact reason why the two groups differed in average age was not known this finding was taken as a given fact and treated as a confounder of the “pesticide-child number issue”. It is evident that with increasing age a man will be able to sire more children. So clearly age is a strong predictor of the number of children. But is the observed difference in the number of children totally explained by the age difference?

### Statistical considerations

In a simple Poisson regression on number of children depending on group and age as a linear variable, the latter has a clear effect with an additional 0.3 children per decade (*p* < 0.001). Even after controlling for age, the exposed group has 0.06 less children but the remaining difference is far from significant. This simplistic approach has two main problems: (a) age is not really linearly associated with number of children but there is an age threshold before men start to sire children and there is likely an age above which no more children are sired. (b) Exposed versus non-exposed was primarily selected on recent exposure; however, reproductive history is, if at all, affected by exposure before conception and therefore by exposures in the past. These two problems were tackled through the following approaches: (a) the shape of the age-child number association was first studied using a spline function (several options for degrees of freedom were applied) in a general additive model (GAM, family: Poisson). On visual inspection of the curves a simpler numerical formula was built (e.g. quadratic term or segmented regression) to mimic the form of the spline. Thereby, the model enabled a better representation of the effect of age but could still easily be interpreted numerically and enhanced by interaction terms. (b) Besides the binary variable of “current exposure yes/no” more detailed data on current exposure (days since last application of pesticides, number of days when pesticides were applied in the last month) were available but deemed irrelevant for past or chronic exposure. For chronic exposure the “number of years in a job with pesticide use” seemed more promising. Exposed workers were asked: “how many years have you been using pesticides?”, while non-exposed workers were asked if they had ever used pesticides for farming. If the answer was affirmative, they were also asked during which years that had happened. Not surprisingly pesticide-years and age were highly correlated rendering models with both factors unstable. Thus “age minus pesticide years” was constructed as a proxy for age at exposure onset because early life exposure, possibly even before reproductive age, was considered most relevant for lifetime reproductive history. For the workers currently not exposed to pesticides but with an exposure history in earlier years their age at exposure onset was extracted from the questionnaires. For the other non-exposed workers the onset age was set equal to their current age. Alternatively “age at onset” was set to an arbitrary constant age in the never exposed group. A priori 90 years was chosen as an age that none of the participants had yet reached. Later on, after studying the upper age threshold of siring children, also that threshold level of 65 years was investigated: because exposure at a very early age was considered detrimental to fathering children, age at onset was also divided into 10-year intervals with the earliest interval ranging from 6 to 15 years. These exposure groups were compared to the group with either no exposure or exposure after the age of 65 years only. In addition, the number of exposure years before the age of 65 years was also examined. Among the controls, only 4 (out of 33) reported use of pesticides ever. Hence calculated age at exposure onset for most of the controls equalled current age (or the fixed age that was set at 90 or 65 years). Thus, among controls, age at calculated exposure onset could have no impact on number of children at all, while age at exposure onset in exposed workers could have. Therefore, it was expected that the age variable would have an effect on the number of children in the whole group, while for the exposure variable the effect would be restricted to the exposed workers only. This was expressed as a Poisson model with an interaction term between group and age at exposure onset and controlling for current age using the best fitting shape of the association. For one pesticide exposed worker information on age was missing. For this worker age was substituted by the average age of all other pesticide exposed workers. In sensitivity analyses this participant as well as the four controls with reported past exposure were dropped.

Finally, analyses were restricted to the exposed workers only: either to all 38 workers currently exposed and the 4 exposed historically, or only the 38 currently exposed workers. Age at onset of exposure, number of exposure years before age of 65 years or age group at onset of exposure were chosen one by one as the independent variable affecting number of children in a Poisson regression. An unadjusted model and a model adjusted for age were compared. All calculations were done with STATA 13.1 SE (StataCorp, College Station, TX, USA).

## Results

**Fig. 1:**
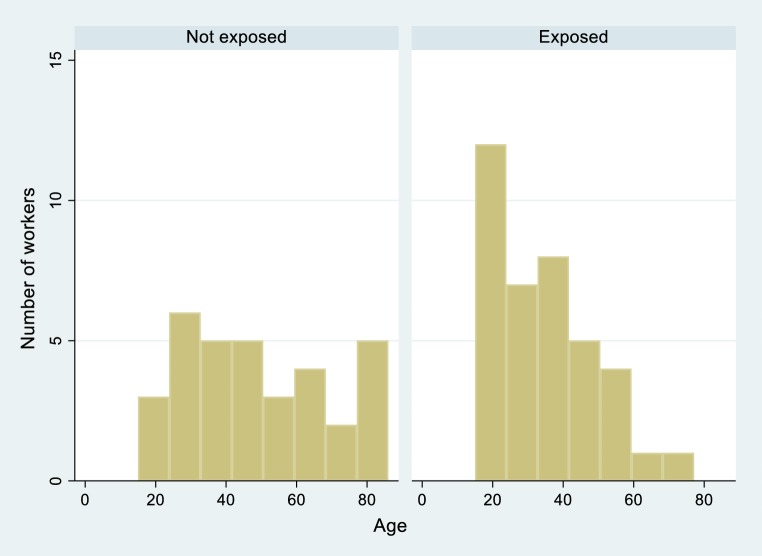
Histograms (per exposure group) displaying the age distribution in exposed and non-exposed participants

Professionally pesticide exposed and non-exposed workers differed in 3 aspects: (i) body weight in controls was somewhat higher (mean ± SE: 71.8 ± 2.1 kg) than in exposed workers (69.1 ± 1.2 kg; *p* = 0.037), (ii) non-exposed participants were older (48.5 ± 3.6 years versus 34.6 ± 2.3 years; *p* = 0.002), and (iii) controls had more children (3.0 ± 0.5, range 0–11 versus 1.6 ± 0.4, range 0–7; *p* = 0.024). The age distribution per group is shown in Fig. 1. Among the organic farming group the age between 20 and 80 years is much more evenly distributed while among the conventional workers young men dominate.

**Fig. 2:**
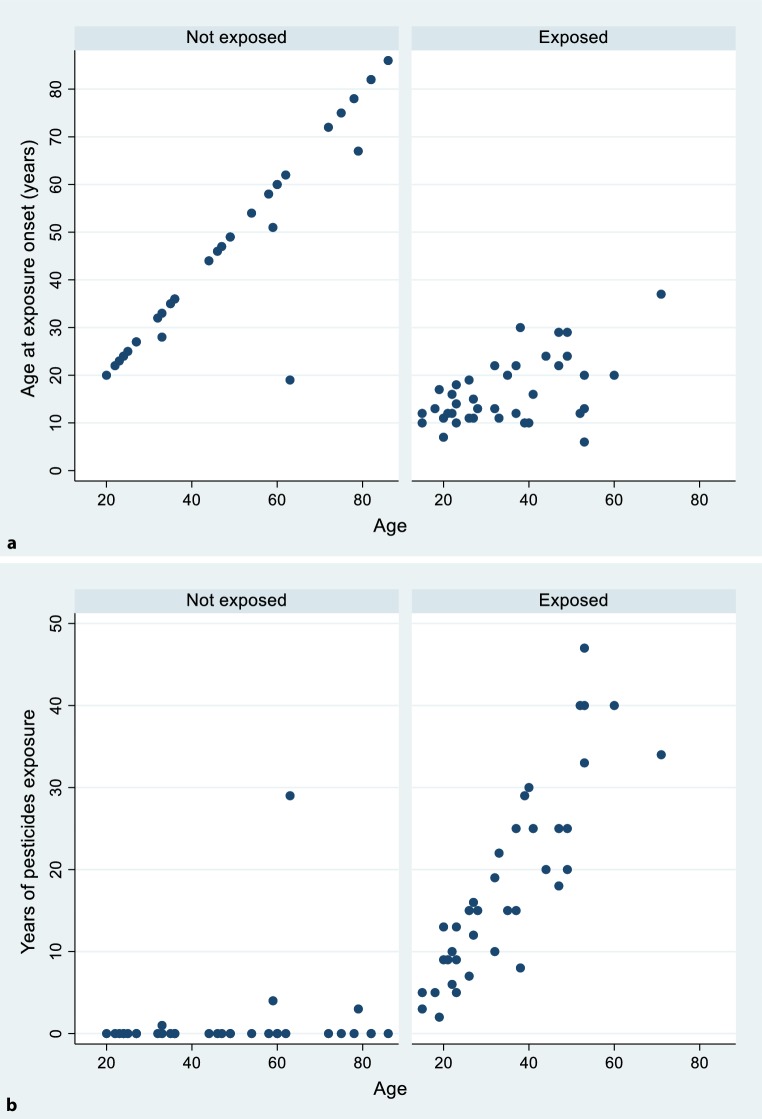
Scatter plots (per exposure group) between age (x-axis) and **a** age at exposure onset or **b** number of exposure years

The number of pesticide years and age at onset of exposure are plotted each against age stratified by group (Fig. 2a, b). Years of education did not differ between groups and was not significantly associated with age or with number of children and thus was not investigated further.

**Fig. 3:**
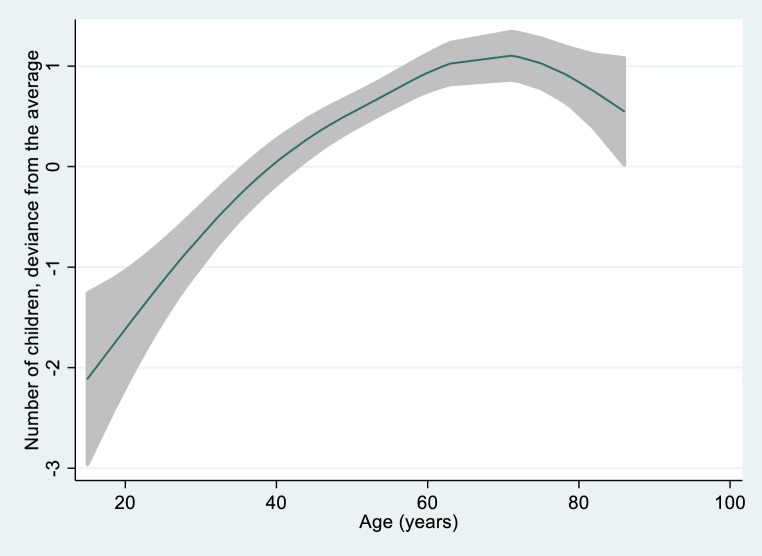
Nonlinear association between age and number of children. Spline function with 3 degrees of freedom

In the GAM of the total sample (71 participants) age displayed an association with number of children that resembled a quadratic function (Fig. 3).

Thus, a quadratic term was built with age squared centered on the maximum of that curve: age squared = (age-65)^2^. Therefore, the linear term of age no longer determined the number of children (*p* = 0.956). The quadratic term was entered into a Poisson regression together with an interaction term between group and age at onset of exposure. A decrease in the number of children above a certain age could be due to a birth cohort effect when the oldest birth cohort experienced socioeconomic or cultural conditions that did not enable them to reap their total fathering potential in earlier years. Nevertheless, an increase in the number of children with increasing age until an upper threshold is reached and after that a constant number of children seems to be biologically more plausible. Therefore, as an alternative to the quadratic term, a broken stick function [27] was built where the cut-off point was determined using the “nl command” in STATA:

$$\mathrm{nl}(\text{children}=\{\mathrm{a}\}* \mathrm{Age}*(\mathrm{Age}<\{\mathrm{b}\})+\{\mathrm{a}\}*\{\mathrm{b}\}*(\mathrm{Age}>=\{\mathrm{b}\})+\{\mathrm{c}\}),$$

where {a} is the slope of the linear regression line below the cut-off, {b} is the cut-off point, and {c} is a constant. In that calculation {b} was estimated as 65.87 years (*p* < 0.001). Consequently 65 years was assumed to be a likely limit for reproduction and a new variable “age until 65 years” was created that equals “age” until 65 years and is 66 when age >65 years. A simple Poisson regression calculating the effect of age until 65 years on the number of children performed nearly equally well (pseudo‑R^2^ = 0.263) as the one using age squared (pseudo‑R^2^ = 0.292). Therefore, the broken stick model was also augmented by the interaction term between group and age at onset.

In the primary model including age squared and the interaction term between group and age at onset, the exposed group had significantly less children (−0.93, *p* = 0.044). Age at onset had no significant effect on number of children in the whole group (*p* = 0.584), but it displayed a significant interaction (*p* = 0.05) with group: in the exposed group each year of earlier exposure reduced the number of children by 0.03 compared to the control group (Table 1). Dropping the single exposed worker with missing information on age did not change the estimates (data not shown) but dropping the 4 control workers with past exposure tended to strengthen the findings (Table 2). When using age until 65 years instead of age squared the results were very similar (data not shown).

**Table 1:** Results of Poisson regression—dependent variable: number of children

Independent variable	Coefficient	5th–95th Confidence interval		*p*-value
Age squared	*−0.00122*	*−0.00157*	*−0.00086*	*<0.001*
Group	*−0.92868*	*−1.83217*	*−0.02519*	*0.044*
Age at onset	−0.00323	−0.01478	0.00832	0.584
Age at onset × group	*0.03102*	*−0.000063*	*0.06210*	*0.050*
Constant	*1.90522*	*1.18393*	*2.62651*	*<0.001*

*Italics* denote significant effects *p* ≤ 0.05

**Table 2:** Poisson regression as in Table 1, but without the 4 control workers with past exposure history

Independent variable	Coefficient	5th–95th Confidence interval		*p*-value
Age squared	*−0.00129*	*−0.0017*	*−0.00089*	*<0.001*
Group	*−1.2761*	*−2.30488*	*−0.24733*	*0.015*
Age at onset	−0.00989	−0.02549	0.00572	0.214
Age at onset × group	*0.03578*	*0.0043*	*0.06726*	*0.026*
Constant	*2.33302*	*1.32469*	*3.34136*	*<0.001*

*Italics* denote significant effects *p* ≤ 0.05

When setting the age at exposure onset for the never exposed workers to 90 years instead to current age an interaction term no longer was reasonable because age at onset was practically a constant in one group. Therefore, a Poisson regression was run with age squared and age at exposure onset. In that model the number of children increased with increasing age at exposure onset (0.03 children per decade); however, this effect did not reach significance (*p* = 0.15). In the professionally exposed only group age at onset was a significant predictor of the number of children but this finding was no longer significant in the adjusted models (Table 3). Likewise, number of exposure years before the year 65 reduced the number of children (0.06 children per 10 years) but the effect was not significant after controlling for age squared (*p* = 0.285).

**Table 3:** Effect estimates of “age at onset” in the exposed only group by adjustment

Adjustment factor	Coefficient	5th–95th Confidence interval		*p*-value
None	*0.07929*	*0.02426*	*0.04864*	*<0.001*
Age squared	0.00957	−0.00488	0.02402	0.194
Age until 65 years	0.00002	−0.01569	0.01574	0.998

*Italics* denote significant effects *p* ≤ 0.05

**Fig. 4:**
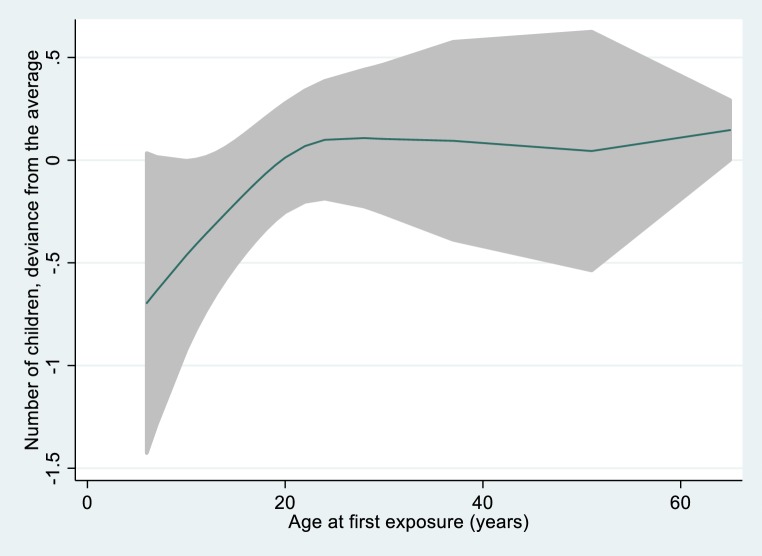
Nonlinear association between age at first exposure and number of children. Spline function with 3 degrees of freedom controlled for current age. For those never exposed or exposed only after an age of 65 years the age at first exposure was set to 65 years

In total 21 participants reported first exposure between 6 and 15 years of age. On the other side 30 participants reported exposure either never or only after the age of 65 years. Even after controlling for age squared in Poisson regression those with early exposure had 0.66 children less (*p* = 0.02) than the late or never exposed ones. In all the other exposure groups the number of children did not differ significantly from that in the comparison group (*p* for trend = 0.102). In a GAM the effect of age and of age at first exposure were both modeled as a spline function (3 degrees of freedom) and the spline for age at first exposure clearly indicated fewer children only when exposure was before an age of around 20 years (Fig. 4). Using the nl command the cut-off point between the rising and the horizontal line was estimated as 22.6 years (*p* < 0.001).

## Discussion

In previous papers [8–10] increased frequencies of cellular abnormalities were demonstrated and symptoms of intoxication with recent exposure to pesticides were reported; however, a reduced number of children was observed in conventional farmworkers that could not be explained by recent exposures. In this study the aim was to investigate better ways to describe pesticide exposure that might be relevant for that endpoint. By repeatedly causing symptoms of intoxication [9] ongoing pesticide exposure could interfere with sexual behavior and thus reduce the number of children with increasing duration of exposure. Through endocrine disrupting effects that are likely most effective during earlier developmental stages, e.g. before or during puberty [28], a one-time or short-time exposure could have a lasting effect on male fertility. In the former case number of years of exposure would be the exposure metric of choice, in the latter case it would be age at first exposure, possibly in relation to puberty. Unfortunately, age is per se a strong predictor of the number of children as well as a predictor of life-long duration of exposure. Controlling correctly for such a strong confounder, especially in the face of a small sample size, is remarkably challenging. This paper also explores ways to solve this problem. Overall the findings indicate that pesticide exposure before the age of about 20 years is an independent predictor of fewer children. This is in accordance with previous experimental [18, 22] and epidemiological [13–17, 19, 29] evidence, even though rats might be even more sensitive than humans [30]. The study did not collect data on types of pesticides used by the participants possibly decades ago. Even pesticides currently in use were so diverse [10] that analysis per type of pesticide was not possible; however, a rough and qualitative list of pesticides without claim for completeness is given: paraquat, glyphosate, pyrethroids, dithiocarbamates, malathion, methamidophos, carbamates, 2,4‑D. For more details refer to the previous study [10]. The study cannot point out a single causal agent, which is a clear limitation. Exposure to pesticides early in life could even be a proxy for e.g. childhood labor and hence for poor socioeconomic conditions; however, better socioeconomic conditions are generally linked to fewer children [31, 32] but at least in reports from Italian agricultural groups an inverse association was noted [33]. In the present data set, highest completed education is the only reliable marker of socioeconomic status. Age is negatively correlated with education (R = −0.4): the only person with a tertiary/university degree was 32 years old. This is also the average age of 18 participants with a secondary school (range: 15–82 years). The same range, but with an average age of 42 years, was found in the 44 persons with compulsory school only. The 8 persons with no schooling at all were on average 49 years old (range: 33–86 years). This is likely due to a birth cohort effect: those born earlier lived in an era when access to schools was less widespread. Although controls were on average older than pesticide exposed workers the education of the two groups did not significantly differ. Among all participants the number of children decreased by 0.3 for each additional step in education (*p* = 0.016) but this association was reversed when controlled for age squared (+0.28 children, *p* = 0.042). Hence poor education that might also be signified by early onset of work could be a possible explanation of the impact of early exposure but the reverse could also be true: early pesticide exposure may be the cause of the association between education and children. Unfortunately, information on age at first work in general is lacking. Therefore, it can only be stated that early work (before about 20 years of age) in conventional coffee farming is associated with fewer children. Exposure to endocrine disrupting pesticides is a plausible but not the only explanation of that finding. Needless to say, current age was treated as an independent factor and a confounder of the association between pesticides and children. As explained in the introduction a causal link between exposure and age cannot be ruled out. In that case controlling for age would not be justified because age then would not be a confounder but an intermediate.

## Conclusion

Fewer children were observed in the group of pesticide exposed workers in comparison to their non-exposed peers. This difference was mostly driven by the age difference between the groups but even after controlling for age a (non-significant) difference remained. Ways to study that difference in spite of small sample size and high correlation between age and exposure indicators were explored. The clearest pesticide effect was seen when first pesticide exposure occurred early in life, i.e. before the age of about 20 years. This is in accordance with endocrine disruptive effects of some pesticides. Direct causal effects are not the only possible explanation of this observation but given the overall scientific evidence, the most likely.
